# Are shoulder surgeons any good at diagnosing rotator cuff tears using ultrasound?: A comparative analysis of surgeon *vs* radiologist

**DOI:** 10.4103/0973-6042.39580

**Published:** 2008

**Authors:** Muthu Jeyam, Lennard Funk, Jonathan Harris

**Affiliations:** Salford Royal NHSFT, Stott Lane, Salford, M6 8HD, United Kingdom; 1 Wrightington Hospital and Manchester Sports Medicine Clinic, 120 Princess Road, Manchester, United Kingdom

**Keywords:** Shoulder, ultrasound

## Abstract

High-resolution ultrasound has gained increasing popularity as an aid in the diagonsis of rotator cuff pathology. With the advent of portable machines, ultrasound has become accessible to clincians.

**Aim:**

This study was conducted to evaluate the accuracy and reliability of ultrasound in diagnosing rotator cuff tears by a shoulder surgeon and comparing their ability to that of a musculoskeletal radiologist.

**Materials and Methods:**

Seventy patients undergoing shoulder arthroscopy for rotator cuff pathology underwent preoperative ultrasonography (US). All patients were of similar demographics and pathology. The surgeon used a Sonosite Micromax portable ultrasound machine with a 10-MHz high frequency linear array transducer and the radiologist used a 9-12 MHz linear array probe on a Siemens Antares machine. Arthroscopic diagnosis was the reference standard to which ultrasound findings were compared.

**Results:**

The sensitivity in detecting full thickness tears was similar for both the surgeon (92%) and the radiologist (94%). The radiologist had 100% sensitivity in diagnosing partial thickness tears, compared to 85.7% for the surgeon. The specificity for the surgeon was 94% and 85% for the radiologist.

**Discussion:**

Our study shows that the surgeons are capable of diagnosing rotator cuff tears with the use of high-resolution portable ultrasound in the outpatient setting.

**Conclusion:**

Office ultrasound, by a trained clinician, is a powerful diagnostic tool in diagnosing rotator cuff tears and can be used effectively in running one-stop shoulder clinics.

Rotator cuff tear is a common disorder that may be debilitating to the patient both in terms of pain and limitation of function.[1] Accuracy in diagnosis and proper management improves the outcome for the patients.

Ultrasonographic evaluation of rotator cuff was first described in 1977.[2] Ultrasound is useful for a dynamic, functional evaluation of the shoulder. It has distinct advantage in that we can perform dynamic assessment and assess the contraction of the muscles too.[3] It is less expensive than MRI, non-invasive, easily available and accurate. It is shown to have high sensitivity and specificity for demonstration of rotator cuff tears.[4–8] However it is operator-dependent and has a learning curve.

In the UK and the US, radiologists mainly perform shoulder ultrasound examinations. Use of office ultrasound by surgeons is slowly gaining popularity. The surgeon has the advantage of a full clinical history and examination of the shoulder and better understanding of the clinical problems of the patient. Thus the surgeons may use the ultrasound as an extension to their clinical examination in an office setting.

In this study we compared the specificity and sensitivity of an Orthopedic surgeon to that of a musculoskeletal Radiologist in diagnosing rotator cuff tears.

## MATERIALS AND METHODS

Seventy consecutive patients undergoing shoulder arthroscopy for rotator cuff pathology underwent preoperative ultrasonography (US). The inclusion criteria were patients with acute or chronic shoulder pain who were referred to the shoulder clinic and who had a high clinical suspicion of rotator cuff tear. The exclusion criteria were patients with learning difficulties and acute fractures. Six patients had not had their arthroscopy at the time the study was completed. Therefore 64 patients were included in this study.

The Shoulder surgeon used a SonoSite Micromaxx portable ultrasound machine, with a high frequency 10 MHz portable linear array transducer (SonoSite Inc. Bothwell, USA), to assess 35 patients. The musculoskeletal radiologist assessed 29 Patients using a 9-12 MHz linear array probe on a static machine (Seimens Antares, Seimens, Germany). Both clinicians had been formerly trained in shoulder ultrasound and had five years of ultrasound experience.

Arthroscopic diagnosis was the reference standard to which ultrasound findings were compared. Standard arthroscopic examination was carried out with careful assessment of the articular and bursal sides of the rotator cuff.

### Ultrasound examination

For the purpose of this study, the senior author (LF) and a musculoskeletal radiologist (JH) carried out the ultrasound examinations. A similar technique was used.

The patient was seated comfortably in a chair, while the examiner either stood behind the patient or sat by the side of the patient. Static and dynamic assessment of the rotator cuff and long head of biceps were done in both transverse and longitudinal planes. Bilateral examinations were carried out if required. Diagnostic criteria for pathology of rotator cuff and long head of biceps were that described by Middleton *et al*.[9] and Teefy *et al*.[6] Full thickness tear was diagnosed when the cuff could not be visualized due to complete avulsion and retraction under the acromion (complete tear) or when there was a focal defect in the cuff created by a variable degree of retraction of the damaged tendons or inversion of the superficial bursa contour. A partial thickness tear was diagnosed when there was a defect on the bursal side of the cuff or a hypoechoic or mixed hyperechoic and hypoechoic defect on the articular side of the cuff. The presence or absence of cuff tear was recorded in detail including the thickness of the tear and the size. All tears were measured using standard ultrasound machine calipers.

At arthroscopy, the presence or absence of full or partial thickness rotator cuff tear was noted. The size of the tear was measured using a linear probe, which had measuring marks 5 mm apart.

### Statistical analysis

A cross table was constructed with the ultrasound assessment against the arthroscopic interrogation of the rotator cuff and biceps tendon [Tables 1 and 2]. Sensitivity and specificity were calculated. Positive and negative predictive values were also extrapolated [Tables 3 and 4].

At arthroscopy, measurements within 5 mm of those obtained by ultrasound were considered correct.

## RESULTS

The results of ultrasound examination both by shoulder surgeon and radiologist corresponded well with arthroscopic findings in 59 of 64 patients, thus giving an accuracy of 92%. The surgeon was able to correctly diagnose full thickness tears with ultrasound in 12 out of 13 patients and the radiologist allowed correct identification in 15 of the 16 patients. As far as the partial thickness tears were concerned the surgeon was able to diagnose 6 of the 7 patients and the radiologist picked up all the partial thickness tears, 6 of 6. There were one false negative and two false positives for the surgeon. The radiologist had one false positive and one false negative. Ultrasound examination by the surgeon demonstrated no tear in 14 of the 15 patients and the radiologist was able to correctly diagnose no tears in 6 of the 7 patients.

**Table 1:** Results of ultrasound diagnosis by the shoulder surgeon

Ultrasound results	Surgical	Findings	
	FTT	PTT	NT
FTT	12	0	1
PTT	0	6	1
NT	0	1	14

FTT - Full thickness tear, PTT - Partial thickness, NT - No tear

**Table 2:** Results of ultrasound diagnosis by the radiologist

Ultrasound results	Surgical	Findings	
	FTT	PTT	NT
FTT	15	0	1
PTT	0	6	0
NT	1	0	6

FTT - Full thickness tear, PTT - Partial thickness, NT - No tear

**Table 3:** Predictive values of ultrasound diagnosis by the shoulder surgeon

	Full thickness tear	Partial thickness tear
Sensitivity	92% (12/13)	86% (6/7)
Specificity	100% (14/14)	93.3% (14/15)
PPV	100% (12/12)	86% (6/7)
NPV	93.3% (14/15)	93.3% (14/15)

PPV - Positive predictive value, NPV - Negative predictive value

**Table 4:** Predictive values of ultrasound diagnosis by the radiologist

	Full thickness tear	Partial thickness tear
Sensitivity	94% (15/16)	100% (6/6)
Specificity	85.7% (6/7)	100% (6/6)
PPV	94% (15/16)	100% (6/6)
NPV	85.7% (6/7)	100% (6/6)

PPV - Positive predictive value, NPV - Negative predictive value

## DISCUSSION

Ultrasound of the shoulder is a reliable, safe and cheap method to diagnose pathology of the rotator cuff and the long head of biceps. Matsen *et al*.[10] have proposed it as a primary mode of imaging for the soft tissues of the shoulder. Iannotti *et al*.[11] have shown in a multi-insttional study that in an orthopedic surgeon's office setting ultrasound can be an effective adjunct to accurate diagnosis of rotator cuff pathology. In addition this may be an opportunity for patient education and explanation of management options.[12] A study by Moosmayer and Smith[13] has shown that orthopedic surgeons with relative inexperience can diagnose accurately rotator cuff pathology using ultrasound.

To our knowledge there has been no study comparing the ultrasonographic skills of an equally trained surgeon and a radiologist. The overall accuracy of the surgeon and the radiologist were comparable at 91.4% and 93%. The sensitivity of ultrasound diagnosis by surgeon and the overall accuracy of the surgeon and the radiologist were comparable at 91.4% and 93%. The sensitivity of ultrasound diagnosis by surgeon and radiologist were comparable for full thickness tears (92% and 94%), however the radiologist were better at diagnosing partial thickness tears with sensitivity of 100% compared to 86% by the surgeon. This could be due to the difference in quality of the machines use, as more subtle pathology is more difficult to discern on current portable machines. Likewise, the lower specificity of the radiologist in this study could be attributed to the higher resolution machine detecting intra-substance tears that are not seen at shoulder arthroscopy. However neither of these factors would have altered the clinical management of the patients.

The specificity for the surgeon was 94% and 85% for the radiologist. We believe this may be due to the surgeon measuring repairable cuff tissue edges at both ultrasound and surgery, whereas the radiologist measures from tendon edges (which is often poor quality cuff tissue).

Our study has shown that shoulder surgeons can effectively perform ultrasound examination of the shoulder. The study also demonstrates the usefulness of ultrasound to the surgeons. Shoulder surgeons who perform ultrasound examination are well suited to run one-stop shoulder clinics, especially with the introduction of ICATS (Integrated Clinical Assessment and Treatment Services).
